# Metasurface-based THz reflectarray antenna with vortex multiplexing and beam-steering capabilities for future wireless communications

**DOI:** 10.1016/j.isci.2022.104725

**Published:** 2022-07-02

**Authors:** Ali Ali, Mohsen Khalily, Tim Brown, Rahim Tafazolli

**Affiliations:** 1 Department of Electrical and Electronic Engineering, 5G & 6G Innovation Centres (5GIC & 6GIC), Institute for Communication Systems (ICS), University of Surrey, Guildford GU2 7XH, UK

**Keywords:** Optical materials, Antennas, Metamaterials

## Abstract

This article investigates the unexplored potentials of vortices or orbital angular momentum (OAM) beams using the low-cost and high-gain dielectric reflectarray antennas (RAs) at the terahertz (THz) band. It proposes a paradigm to enable 3D beam-steering or OAM multiplexing by a single structure via tilted OAM beams. That, in turn, requires reaching the maximal attainable angles either to send multiple beams to different receivers or to focus the OAM beams of different modes in a desired direction. For this reason, two concepts are addressed in this work: (i). generating a single 3D steered OAM beam and (ii) producing multiple off-centered OAM beams with different modes. A volumetric unit cell is adopted to be accurately tuned through the aperture to steer the generated beams towards the desired direction(s). The proposed paradigm can be utilized to produce RAs with beam-steering or OAM multiplexing capabilities as candidates for THz indoor communications.

## Highlights

• Employing tilted OAM beams at the terahertz band for wireless communications
• Applying OAM multiplexing using a passive reflectarray antenna
• Producing multiple off-centered OAM beams with different modes

## Graphical abstract

**Figure:**
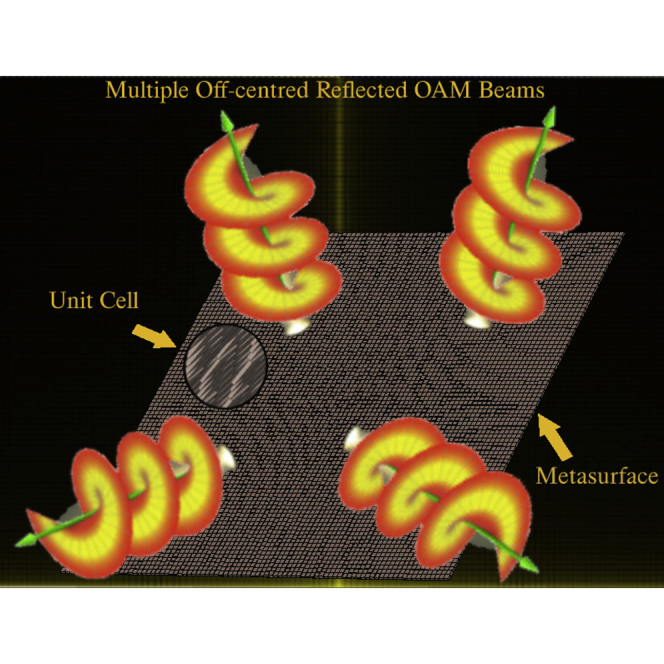

## Introduction

The dramatic increase in the usage of online services during the Covid-19 pandemic, the ever-growing internet demand, and the ultra-high requirements of the fifth generation (5G) networks form a critical necessity to accelerate the development of wireless communication systems. To bear the burden of the multiple gigabits per second (Gbps) data rates expected within the next few years, expanding the application, transport, and network layers' capacities of communication systems would be inevitable (D. Loghin et al., 2020).

One of the solutions nominated for the physical layer is employing the spatial distribution of the electromagnetic (EM) wave during its propagation, which can be acquired by designing angular momentum (AM)-based structures. Given that AM has two components, the spin angular momentum (SAM) and orbital angular momentum (OAM). OAM-based structures can allow the simultaneous data exchange of several OAM modes through the same frequency (D. Berry et al., 1963) without occupying more frequencies from the available spectrum owing to the inherited orthogonality among its modes. Several methods have been discussed to produce OAM beams, such as the spiral phase plates (SPPs) (B. Allen et al., 2019), dielectric resonators (DR) (Y. Pan et al., 2015), and the annular parabolic trough reflector array (H. Wang et al., 2016). Most of the published works about generating OAM beams demonstrated producing a single mode per structure, which raised concerns about the ability of OAM waves to enhance the channel capacity and spectral efficiency or not. Undoubtedly, the main interest in employing the OAM beams is to exchange data simultaneously via OAM multiplexing. Accordingly, single-mode structures cannot have a significant impact on improving the throughput of wireless communication systems. Specifically, most of the forenamed designs could not apply the OAM multiplexing among different modes.

The OAM multiplexing is usually achieved by generating different concentric modes that are stemmed from the same point (Lee et al., 2019). In that case, active elements are requisite, which will lead to pricey and complex designs as feeding networks are compulsory. It should be noted that the feeding network complexity is proportional to the number of the needed OAM modes. To avoid that, generating multiple off-centered beams of different modes can be used to perform the OAM multiplexing by passive structures. Consequently, modifying the beam center and flexibly controlling the beam direction would be critical to focus the beams towards the same receiver. Although several articles have discussed producing OAM beams with controlled angles (Huang and Li, 2020; Li et al., 2021) together with multiple OAM beams of different modes (S. Yu et al., 2016; D. Zhang et al., 2018), they could not provide a concrete solution to the OAM problems. As the produced beams were originated from the same point, OAM beams were restricted to a single direction either along the x or y-axis, and the maximum achieved angles were small that did not exceed 40°.

However, embracing a new paradigm, like the transition from linear momentum-based structures to orbital angular momentum-based ones, demands adequate time to be studied, simulated, and practically investigated. Hence, initial designs are discussed even though they do not fully meet all the desired conditions but can be gradually developed to fulfill them. To correctly utilize the features of OAM beams, this article presents a design with a three-dimensional (3D) flexible control on the direction of the beam and a new method to produce multiple off-centered OAM beams of different modes using a single feeder. To the best of the authors' knowledge, this is the first work suggesting OAM multiplexing using a passive structure. Here, different scenarios of OAM beams are addressed in terms of the azimuth angle θ, elevation angle φ, number of generated beams, and mode number l to verify the aimed goals as follows:

• A single tilted OAM beam with (l=1, θ=45° & φ=45°).
• Four steered off-centered OAM beams with different modes: (l=1, θ=20° & φ=−20°), (l=2, θ=−20° & φ=20°), (l=3, θ=−20° & φ=−20°), and (l=4, θ=20° & φ=20°).
• Four off-centered tilted OAM beams with different modes of different angles toward a single receiver for OAM multiplexing enabling: (l=1, θ=−20° & φ=−20°), (l=2, θ=20° & φ=20°), (l=3, θ=20° & φ=−20°), and (l=4, θ=−20° & φ=20°).

The prior scenarios will be validated by virtue of simulations as the fabrication at 330 GHz was not possible at this juncture.

However, the excellent ability to control the phase-magnitude characteristics and the high gain has made metasurfaces a tailored fit candidate to shape OAM beams (Z. Y. Yu et al., 2021; Li and Chen, 2021). That has been broadly demonstrated in the literature (Lei and Chang, 2017). A reflectarray antenna (RA) is an example of metasurfaces that can reflect the incident EM wave and regulate the phase of the incident beam as they combine the advantages of parabolic reflectors and phased array antenna characteristics in one structure (S. Montori et al., 2015). Although the low profile, low cost, and high gain of the RAs have encouraged employing them in various applications, the inherited narrow bandwidth is still the key concern related to choosing RAs (Imaz-Lueje et al., 2020). Although frequency reuse (FR) and other techniques have been addressed in the literature as solutions to overcome this downside of RAs (Martinez-de-Rioja et al., 2016; Nayeri et al., 2011), utilizing OAM can increase spectral efficiency. Nevertheless, the thirst for higher bandwidth has resulted in embracing higher frequencies to overcome this issue by exploiting the vast unexplored bandwidth as the terahertz (THz) gap from 100 GHz to 10 THz (Alibakhshikenari et al., 2021). Also, the high-penetration capabilities (R. Deng et al., 2014) and the non-ionizing property (Mukherjee and Gupta, 2008) are additional features of the THz band. Utilizing the THz spectrum is not a straightforward process where the coverage, fabrication costs, and propagation losses should be considered (Alibakhshikenari et al., 2020). As it is known, the current spectrum allocation of active services stops at 275 GHz (Serghiou et al., 2022) and the undesirable molecular absorptions can significantly affect the propagation losses at higher frequencies, particularly water vapor begins above 300 GHz (Chen et al., 2021). Accordingly, a frequency of 330 GHz is chosen for the proposed designs. To avoid the power losses resulting from the resonant patches at THz, which can reduce the phase range of the unit cell, the dielectric unit cell is employed in this work (P. Nayeri et al., 2014).

This contribution solves for the first time two critical challenges related to OAM beams. Firstly, it highlights the capability of controlling the direction of OAM beams at the THz band to achieve the required line-of-sight (LoS) for multiple beams of different modes. Furthermore, it proposes a novel design with OAM multiplexing without PIN diodes and varactors, which would be very expensive and complex to be built at 330 GHz. Both designs can play considerable roles in the development of the physical layer of the current wireless networks.

## Reflectarrays design and analysis

**Figure 1:**
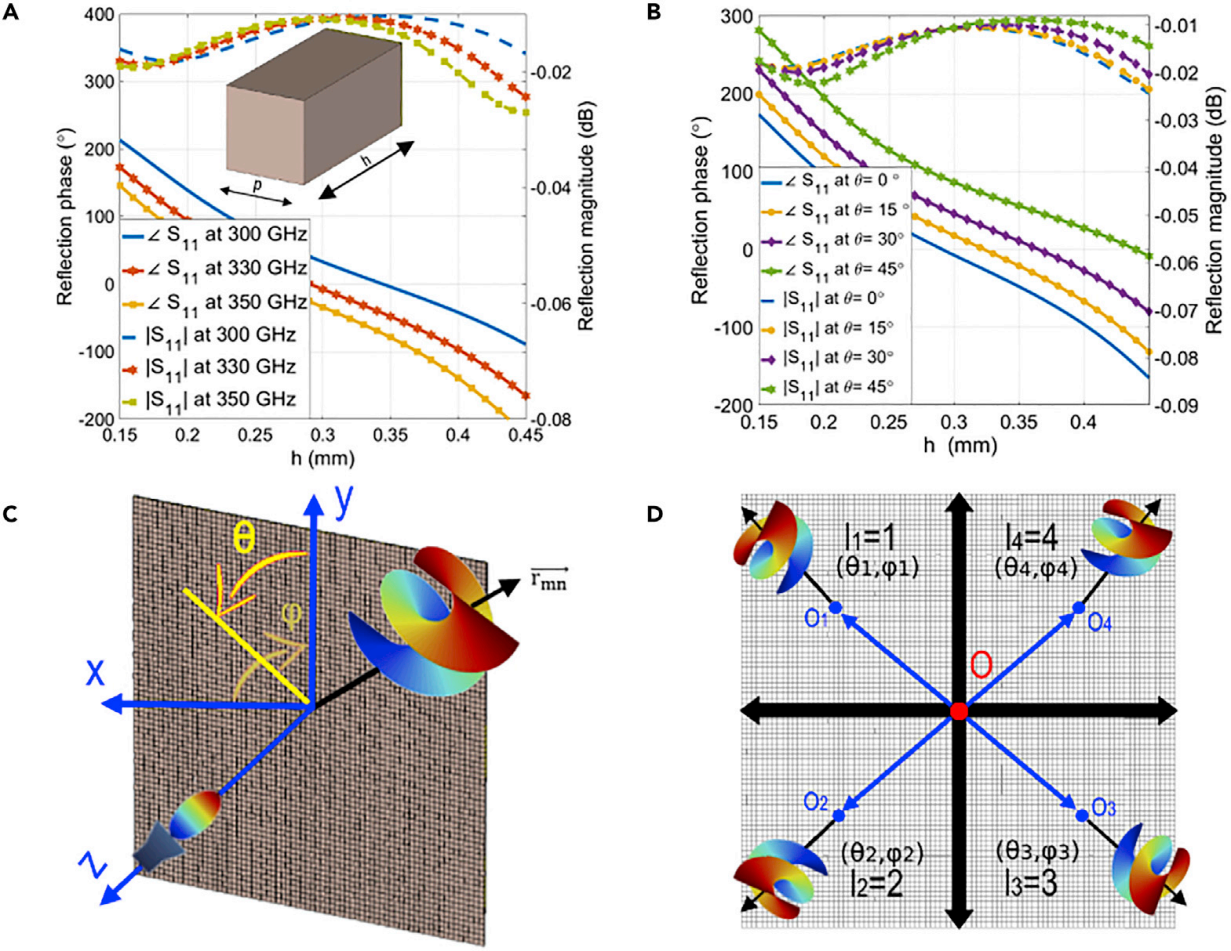
Unit cell specifications and configurations of single and multiple tilted OAM beams (A) Geometry and reflection responses of the column-type unit cell (B) Unit cell response to oblique angle incidences (C) Configuration of tilted OAM generation (D) Configuration of multiple tilted off-centered OAM beams of different modes.

Three single-layer RAs of 82 × 82 column-type dielectric unit cells will be studied in this article. The period of the proposed unit cell is $p=\lambda_{0}/2=\;$ 0.45 mm, the relative permittivity of the substrate is $\varepsilon_{r}=$ 10, and the thickness variation of the RAs spans between 0.8 and 1.1 mm to reach a sufficient phase range to produce the desired beam (Nayeri et al., 2018). As shown in (Figure 1A), a reflection phase of over 300° and reflection magnitude of lesser than −1 dB at 330 GHz are achieved.

As it is known, the phase distribution of the elements over the RAs' aperture should follow the shape of the desired beam. To generate a pencil beam, a specific phase distribution would be required that mimics several concentric circles to produce a spherical wavefront. The phase variation over an aperture of m×n elements can be expressed by the following equation (Shaker et al., 2014):

(Equation 1) $${\text{ψ}}_{mn}=k\left(R_{mn}-{\vec{r}}_{mn}.{\hat{u}}_{b}\right)+{\text{ψ}}_{0}$$

where k is the free space wavenumber, the distance between a specific unit cell mn and the phase center of feeder is represented by $R_{mn}$, ${\hat{u}}_{b}$ stands for the beam direction, ${\vec{r}}_{mn}$ represents its vector, and ${\text{ψ}}_{0}$ is a constant phase.

As for the phase distribution needed to produce helical wavefronts for single and multiple steered OAM beams like the shown in (Figures 1C and 1D), it will be thoroughly discussed in the following subsections:

### Single 3D steered orbital angular momentum beam

It is crucial to investigate the maximum angles that tilted OAM waves can reach by planar structures such as RAs. The proposed design will not only extend the steered angles beyond 30° but also will control two angles simultaneously to attain a 3D beam-steering. However, by finding the solution of Helmholtz’s equation in the circular cylindrical coordinate system, the electric field of *l*-order Bessel beam can be expressed as:

(Equation 2) $$E\left(\text{ρ},\text{φ},z\right)=E_{0}\cdot J_{l}\cdot \left(k_{\text{ρ}}\cdot \text{ρ}\right)\cdot e^{-j.k_{z}.z}\cdot e^{-j.l.\varphi }$$

where $J_{l}$ represent the Bessel function of the order l. $k_{\rho }$ and $k_{z}\;$ are the components of the wave-vector in the circular cylindrical coordinates. It is apparent that the electric field of the Bessel beam includes $e^{-j.l.\varphi }$, which reveals that Bessel beams can support OAM.

Accordingly, to produce an OAM beam, the component $e^{-j.l.\varphi }$ should be added to the phase value shown in Equation (1). Moreover, to steer the generated beam in a specific direction like the beam in (Figure 1C), ${\hat{u}}_{b}$ and ${\vec{r}}_{mn}$ should be characterized by angles (θ, φ). Therefore, the required phase for tilted OAM can be described as:

(Equation 3) $${\text{ψ}}_{m,n}=k\cdot \left(R_{mn}-\sin\text{θ}\cdot \left(x_{m}\cdot \cos\text{φ}+y_{n}\cdot \sin\text{φ}\right)\right)+l\times \arctan\left(y_{n}/x_{m}\right)+{\text{ψ}}_{0}$$

where ($x_{m}$, $\;y_{n}$) denote the element’s position.

**Figure 2:**
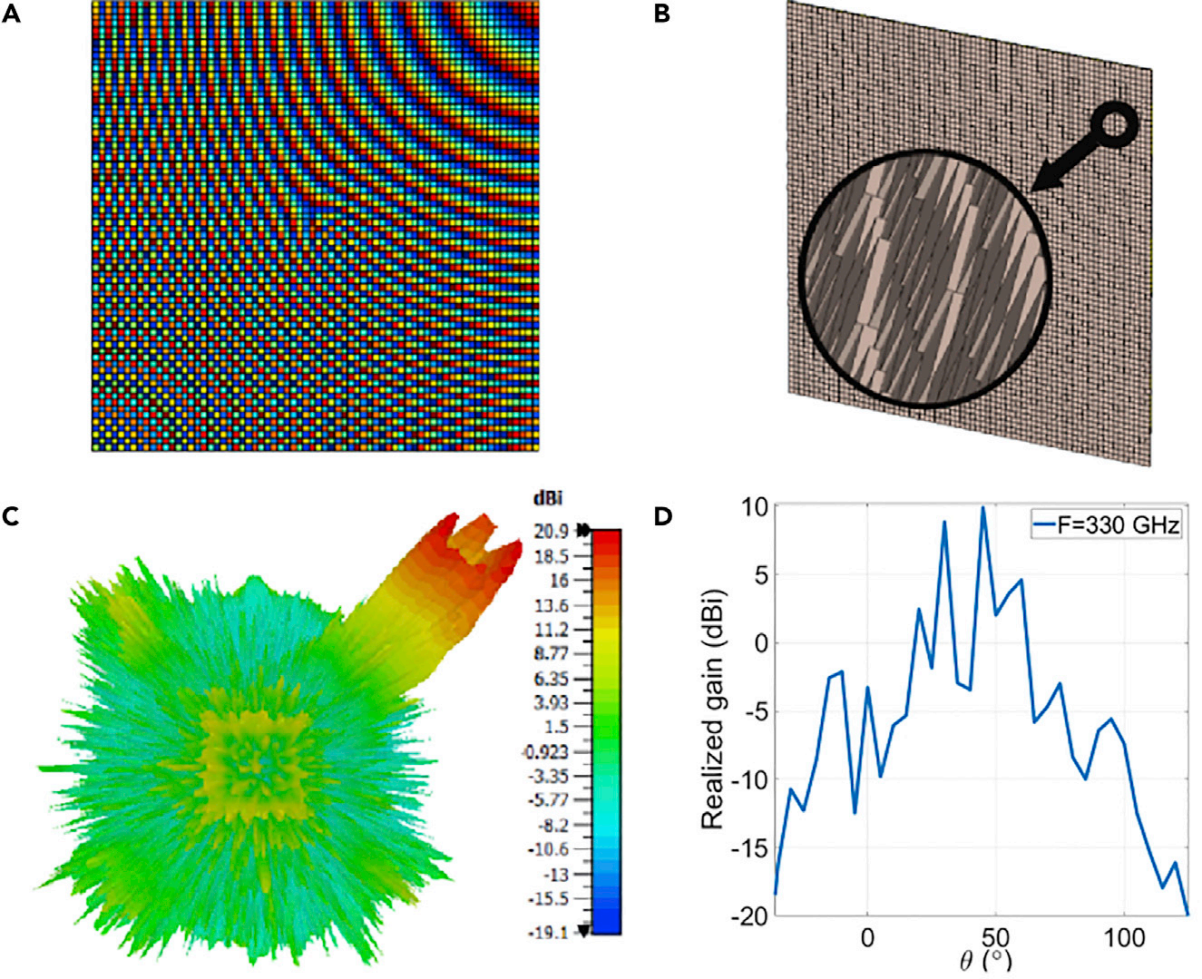
Simulation results of tilted OAM RA with θ= 45° & φ= 45° with l= 4 at 330 GHz (A) spiral phase distribution (B) RA layout (C) radiation pattern (D) realized gain.

The needed phase distribution to produce a tilted OAM beam with maximum degrees θ= 45° & φ= 45° is calculated by Equation (3) and illustrated in (Figure 2A). The thickness variations of unit cells follow the previous phase delay distribution. The 37 × 37 mm^2^ RA shown in (Figure 2B) illustrates the changes in the thickness through the whole aperture, which is obtained by CST Studio Suite 2021 with the periodic boundary condition and Floquet excitation. Simulation results show a null in the radiation pattern as displayed in (Figures 2C and 2D). The phase and magnitude of the electric field cannot be observed by normal cutting planes. Therefore, transformation matrices should be utilized to rotate the corresponding plane based on the values of θ and φ.

As the generated beams by metasurfaces can be controlled by two angles as depicted in (Figure 1C) where it illustrates the rotation ranges of θ and φ along x and z, respectively. That means $R_{x}\left(\alpha \right)$ and $R_{z}\left(\gamma \right)$ in the transformation matrices should be applied to rotate the cutting plane so the E-field characteristics can be observed for the tilted beams. The resulted matrix for the beam with θ = 45° & φ= 45° can be expressed as:

$$R=\left[\begin{array}{lll} 0.71 & -0.71 & 0\\ 0.5 & 0.5 & -0.71\\ 0.5 & 0.5 & 0.71 \end{array}\right]$$

**Figure 3:**
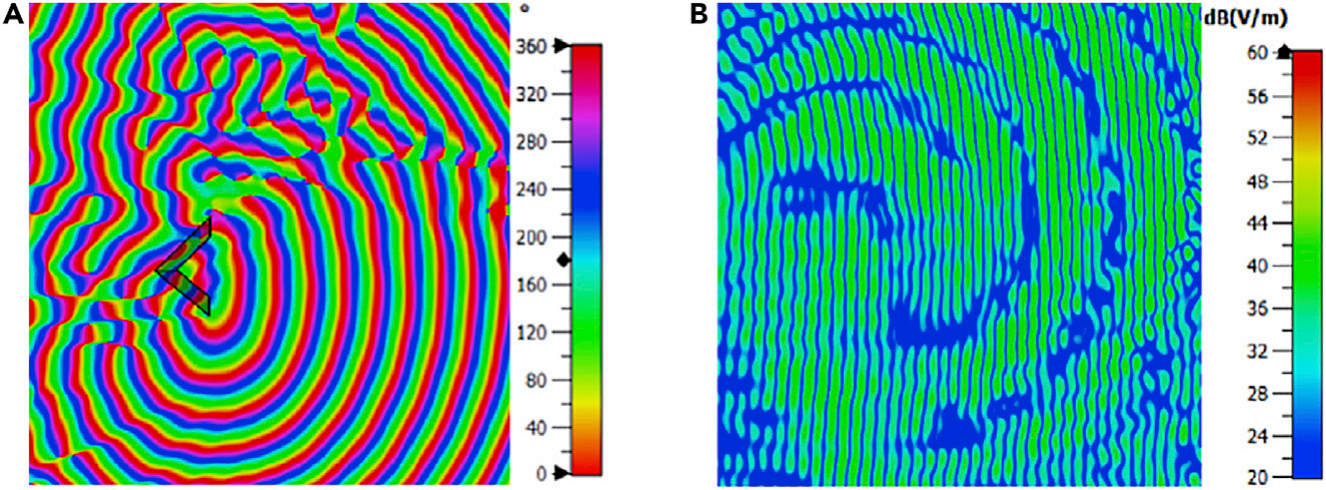
Simulated phase and amplitude distributions on the scanning plane Electric field of the tilted OAM beam of \theta = 45° & \varphi = 45° with l = 4 at 330 GHz at 330 GHz (A) Phase distribution (B) Magnitude distribution.

The spiral phase distribution can be noticed in (Figure 3A) along with the intensity null in the magnitude distribution of the E-field shown in (Figure 3B). Depending on these results, the generation of tilted OAM beam with the maximum achievable angles of planar shape is confirmed for l= 4 at 330 GHz.

### Multiple off-centered orbital angular momentum beams of different modes

The previous attempts to generate multiple OAM beams could not yield real solutions for practical applications as they were limited to 1D steering capability on the beam direction. Also, the produced beams were originated from the same center. Consequently, it was not possible to attain OAM multiplexing and flexible beam-steering. The solution suggested in this article is generating off-centered multiple tilted OAM beams of different modes per a single feeder. Therefore, the effect of oblique incidences should be considered for the proposed unit cell to avoid feed-to-metasurface mismatch. As illustrated in (Figure 1B), the variations in the angle of incidence have a negligible impact on the phase and magnitude of the unit cell. Here, the introduced concept depends on finding new origin points instead of one main center as exhibited in (Figure 1D), each of them would be related to a single OAM beam. At the same time, the needed area to satisfy the phase delay distribution to generate OAM beams is reduced. That will result in generating low divergence OAM beams. Usually, to generate a single beam of a single mode, Equation (3) is applied. Whereas in the case of generating four beams of different OAM modes with the exact dimension of the metasurface with a single beam, the following general equation should be employed:

(Equation 5) $${\text{ψ}}_{m_{k},n_{k}}=k\cdot \left(R_{m_{k}n_{k}}-\sin\text{θ}\cdot \left(x_{m_{k}}\cdot \cos\text{φ}+y_{n_{k}}\cdot \sin\text{φ}\right)\right)+l_{p}\times {\text{ψ}}_{k}+{\text{ψ}}_{0}$$

where k= 1,2,3,.. represents the number of generated beams and p= 1,2,3,.. is the mode number of corresponding OAM beam. As generating four beams require new centers $O_{k}\left(x_{m_{k}},y_{n_{k}}\right)$, the following equation will calculate the phase delay distribution of each beam depending on its allocated area as follows:

(Equation 6) $${\text{ψ}}_{k}=\arctan\left(\left(\left(y_{o}\pm d/4\right)-y_{n_{k}}\right)/\left(\left(x_{o}\pm d/4\right)-x_{m_{k}}\right)\right)$$

where d is the length of the antenna’s side (square-shape) or the diameter (circular shape) and $O\left(x_{0},y_{0}\right)$ represents the main origin point.

In the following subsections, the previous concept will be employed to generate multiple tilted OAM beams of different modes for beam-steering and multiplexing applications as follow:

### Orbital angular momentum beam-steering

Here, generating tilted OAM beams for beam-steering applications is discussed through 37 × 37 mm^2^ single layer RA at a frequency of 330 GHz. The first step is to generate the needed phase delay distribution to arrange the elements based on the four centers and directing the beams towards θ = ±20° & φ= ±20°. For the beam-steering case, Equation (5) will be extended to the following equations:

(Equation 7) $${\text{ψ}}_{m1,n1}=k\cdot \left(R_{m1n1}-\sin\left(\text{θ}\right)\cdot \left(x_{m1}\cdot \cos\left(-\text{φ}\right)+y_{n1}\cdot \sin\left(-\text{φ}\right)\right)\right)+l_{1}\times {\text{ψ}}_{1}+{\text{ψ}}_{0}$$

(Equation 8) $${\text{ψ}}_{m2,n2}=k\cdot \left(R_{m2n2}-\sin\left(-\text{θ}\right)\cdot \left(x_{m2}\cdot \cos\left(\text{φ}\right)+y_{n2}\cdot \sin\left(\text{φ}\right)\right)\right)+l_{2}\times {\text{ψ}}_{2}+{\text{ψ}}_{0}$$

(Equation 9) $${\text{ψ}}_{m3,n3}=k\cdot (R_{m3n3}-\sin\left(-\text{θ}\right)\cdot \left(x_{m3}\cdot \cos\left(-\text{φ}\right)+y_{n3}\cdot \sin\left(-\text{φ}\right)\right))+l_{3}\times {\text{ψ}}_{3}+{\text{ψ}}_{0}$$

(Equation 10) $${\text{ψ}}_{m4,n4}=k\cdot \left(R_{m4n4}-\sin\left(\text{θ}\right)\cdot \left(x_{m4}\cdot \cos\left(\text{φ}\right)+y_{n4}\cdot \sin\left(\text{φ}\right)\right)\right)+l_{4}\times {\text{ψ}}_{4}+{\text{ψ}}_{0}$$

**Figure 4:**
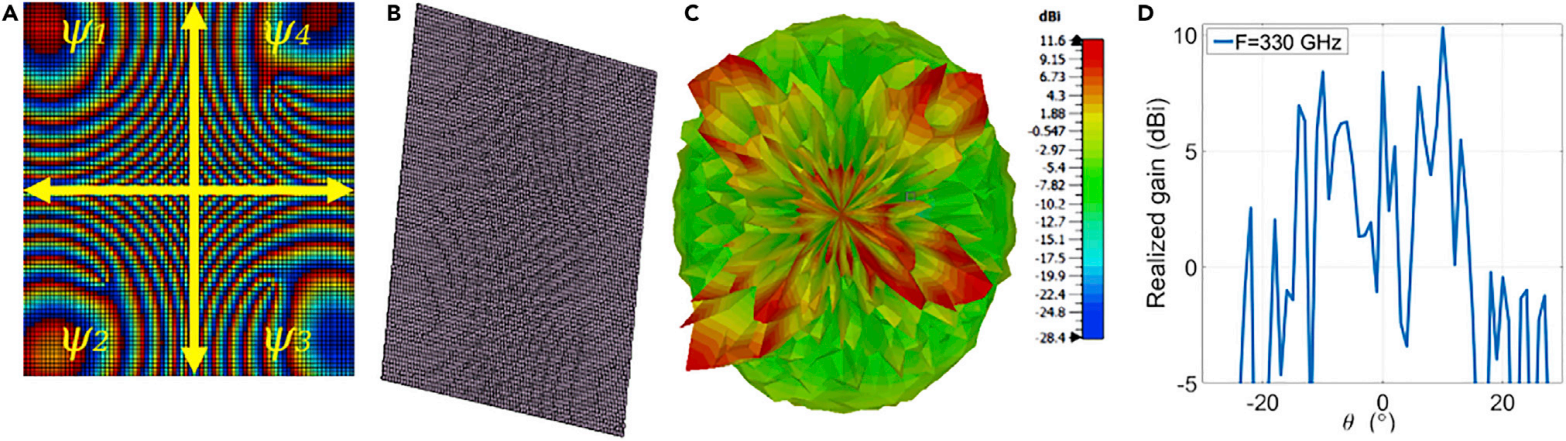
Simulation results of multiple tilted OAM beams for the modes \ l = 1, \ l = 2,\ l = 3, and \ l = 4 at 330 GHz with \theta = ±20° & \ varphi = ±20° at 330 GHz (A) spiral phase distribution (B) RA layout (C) radiation pattern (D) realized gain.

The phase delay distribution is needed to generate the four tilted beams per single feeder for a normal incidence-centered feeder, shown in (Figure 4A). It is calculated by substituting Equation (6) in Equation (5) for each beam. This phase is utilized to build the RA illustrated in (Figure 4B). As the changing parameter is the thickness of unit cells, the front view cannot clearly show the phase distribution. For this reason, it is demonstrated in an orthographic view. It is evident in the radiation pattern shown in (Figure 4C), four tilted OAM beams of different modes with θ = ±20° & φ= ±20° are generated with a high gain as demonstrated in (Figure 4D) at a frequency of 330 GHz.

### Orbital angular momentum multiplexing

The introduced design produces four steered OAM beams towards a specific direction (receiver) by controlling the values of θ & φ to enable the OAM multiplexing at a frequency of 330 GHz. For example, if the receiver is at a specific location A(x,y,z) from the Metasurface, each couple of $\left(\theta_{1},\varphi_{1}\right)$, $\left(\theta_{2},\varphi_{2}\right)$, $\left(\theta_{3},\varphi_{3}\right)$, and $\left(\theta_{4},\varphi_{4}\right)$ would be calculated to steer the beam generated from the associated sub-Metasurface towards that direction. That means the produced structure will employ the off-centering technique to release the multiple beams from the restrictions of the main origin point. Thus, the multiplexing feature can be obtained by a passive structure. The design later in discussion demonstrates employing OAM multiplexing for a receiver located at the normal of the Metasurface. Then, Equation (5) will be extended to the following equations:

(Equation 11) $${\text{ψ}}_{m1,n1}=k\cdot \left(R_{m1n1}-\sin\left(-\text{θ}\right)\cdot \left(x_{m1}\cdot \cos\left(-\text{φ}\right)+y_{n1}\cdot \sin\left(-\text{φ}\right)\right)\right)+l_{1}\times {\text{ψ}}_{1}+{\text{ψ}}_{0}$$

(Equation 12) $${\text{ψ}}_{m2,n2}=k\cdot \left(R_{m2n2}-\sin\left(\text{θ}\right)\cdot \left(x_{m2}\cdot \cos\left(\text{φ}\right)+y_{n2}\cdot \sin\left(\text{φ}\right)\right)\right)+l_{2}\times {\text{ψ}}_{2}+{\text{ψ}}_{0}$$

(Equation 13) $${\text{ψ}}_{m3,n3}=k\cdot \left(R_{m3n3}-\sin\text{θ}\cdot \left(x_{m3}\cdot \cos\left(-\text{φ}\right)+y_{n3}\cdot \sin\left(-\text{φ}\right)\right)\right)+l_{3}\times {\text{ψ}}_{3}+{\text{ψ}}_{0}$$

(Equation 14) $${\text{ψ}}_{m4,n4}=k\cdot \left(R_{m4n4}-\sin\left(-\text{θ}\right)\cdot \left(x_{m4}\cdot \cos\left(\text{φ}\right)+y_{n4}\cdot \sin\left(\text{φ}\right)\right)\right)+l_{4}\times {\text{ψ}}_{4}+{\text{ψ}}_{0}$$

**Figure 5:**
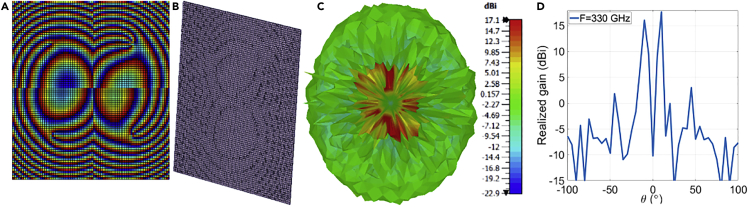
Simulation results of OAM multiplexing for the modes \ l = 1, \ l = 2,\ l = 3, and \ l = 4 at 330 GHz with \theta = ±20° & \varphi = ±20° at 330 GHz (A) spiral phase distribution (B) RA layout (C) radiation pattern (D) realized gain.

Bearing in mind that all the metasurfaces discussed in this work have the same size, which is 37 × 37 mm^2^ (Figure 5B). demonstrates the layout of the designed RA after following the phase delay distribution exhibited in (Figure 5A). The OAM multiplexing is confirmed in (Figures 5C and 5D) where the intensity null is evident at the center of the radiated beams. A comparison between the multiple-tilted OAM beams produced by the introduced metasurface and other structures reported in the literature is given in (Table 1). It is clear from the table that the suggested structure delivers 3D multiple beams of different modes of orbital angular momentum that are capable to perform beam-steering/multiplexing compared to the designs in literature.

**Table 1:** Comparison between the multiple OAM beams presented in this design and the reported in the literature

Ref.	Size [mm]	Frequency [GHz]	Gain [dBi]	Beam-steering/Max. angle	Number of beams/number of modes	Fractional bandwidth	Multiplexing capability
[7]	10.24×λ	20	20.3	2D/ θ=|30| °	4/2	–	N
[8]	106.7×λ^a^	1000	–	3D/ θ=|21| °	4/4	–	N
[9]	9.4×λ	5.6	17	2D/ θ=|30| °	2/2	16%	N
[10]	8.8×λ	8.5	–	2D/ θ=|40| °	4/4	–	N
This work	40.7× λ	330	20.9	3D/ θ=|45| °	4/4	15%	Y

a In this work, the authors mentioned the size of the structure as 32 × 32 elements. The element size was not mentioned, we assumed it as (λ_0_/2) to find the approximate size.

### Conclusion

This article discussed some of the unexplored potentials of OAM beams through reflectarray antennas at 330 GHz. It investigated the maximum achievable angles by a planar metasurface per single feed for a single OAM beam. That motivated the proposed work to examine the possibility of generating multiple off-centered OAM beams of different modes with the maximal achievable angles for OAM multiplexing and beam-scanning applications. Simulation results provided encouraging results for all the discussed scenarios. The designed RAs can be envisaged for THz indoor communications.

### Limitation of the study

The fabrication of structures at 330 GHz is still the key challenge owing to the extraordinarily high accuracy needed, especially for building dielectric structures with variable thicknesses.

## STAR★Methods

### Key resources table

**Table:** 

REAGENT or RESOURCE	SOURCE	IDENTIFIER
**Software and Algorithms**		

CST Studio Suite 2020	Dassault Systèmes	https://www.3ds.com

### Resource availability

#### Lead contact

Further information and resources related to this study will be fulfilled by the lead contact Ali Ali (ali.ali@surrey.ac.uk) upon reasonable request.

#### Materials availability

This paper did not generate new unique reagents.

### Method details

#### Orbital angular momentum generation

The geometry of the unit cell is selected and simulated to achieve a reflection phase range of 300° or above. Then the phase distribution of OAM was obtained by adding the component to change the spherical wavefront generated by Equation (1) so that the wave carried angular momentum. Next, the structure was built in CST Suite Studio and simulated. The presence of phase null in the magnitude of the electric field and the spiral phase distribution of the phase of the electric field confirmed the generation of OAM beams.

#### Quantification and statistical analysis

Metrics are given and this could be verified in the responses of the proposed structures.

## Data Availability

This paper does not report data/code.
